# Development and Validation of a Symptom Scale for Indian Patients With Medically Unexplained Physical Symptoms

**DOI:** 10.7759/cureus.55956

**Published:** 2024-03-11

**Authors:** Aakansha, Amandeep Singh, Piyush Ranjan, Tanveer Kaur, Nandini Rawat, Siddharth Sarkar, Gauri Shanker Kaloiya, Anamika Sahu, Koushik Sinha Deb, Upendra Baitha

**Affiliations:** 1 Medicine, All India Institute of Medical Sciences, New Delhi, IND; 2 Psychiatry, All India Institute of Medical Sciences, New Delhi, IND; 3 Clinical Psychology, All India Institute of Medical Sciences, New Delhi, IND

**Keywords:** india, symptom severity, scale development, construct validity, mups

## Abstract

Introduction

Patients with medically unexplained physical symptoms (MUPS) account for a substantial proportion of patients visiting the outpatient department. Diagnosis of MUPS is a challenge for most physicians. An accurate diagnosis relies on obtaining a detailed history from patients regarding the nature of their symptoms, their onset, and any associated aggravating or relieving factors. This study aims to develop a symptom scale for Indian patients with MUPS.

Methods

The study had a mixed-method study design. Phase 1 involved designing the questionnaire using qualitative techniques, such as literature reviews, focus-group discussions, expert evaluation, and pre-testing of a Hindi and English language Likert-rated interviewer-administered scale. In phase 2, the construct validity of the questionnaire was established using quantitative approaches among 116 patients diagnosed with MUPS.

Results

The final questionnaire consists of 38 items, with good internal consistency (Cronbach 𝛂 = 0.916). Confirmation sampling adequacy for factor analysis was done using the Kaiser-Meyer-Olkin test (KMO value = 0.792) and Bartlett’s test of sphericity (p < 0.001). The newly developed scale showed a Pearson correlation coefficient of 0.568 (p < 0.001) with Patient Health Questionnaire (PHQ)-15 scores.

Conclusion

A reliable and valid tool has been developed to assess patients’ symptoms with MUPS in English and Hindi languages. This questionnaire can be used for assessment, screening, and diagnostic purposes as well as to chart longitudinal changes in patients with MUPS.

## Introduction

Medically unexplained physical symptom (MUPS) is an umbrella term for physical symptoms with little or no identified medical cause. According to a study conducted in the medicine outpatient department at a hospital in India by Baitha et al., the prevalence of MUPS was 24.6% among patients aged 18-60 years, with a slight female preponderance [1,2].

The diagnosis of MUPS relies on the absence of identifiable medical explanations despite thorough evaluation, making it a diagnosis of exclusion. Medical history and clinical features aid in diagnosing specific diseases, but in the case of MUPS, they primarily help exclude explainable medical illnesses. In different cultures, patients present with different symptoms, and the ability of subjects to differentiate between psychogenic and medical symptoms may be affected by the practices of local physicians. Patients also have their own culturally supported theories for the etiology of their symptoms [3,4].

Several instruments are available at present for the diagnosis of MUPS, most of which have been developed for patients from Western countries, and even though translated versions of these scales exist, their cultural validity remains questionable. Hence, there is a need to develop a scale based on local data to help us better understand our patients presenting with MUPS. The purpose of this study is to address this significant constraint by developing a user-friendly symptom scale for Indian patients diagnosed with MUPS, which can be easily administered, takes minimal time and expertise, and is accessible in Hindi and English languages.

## Materials and methods

**Figure 1:**
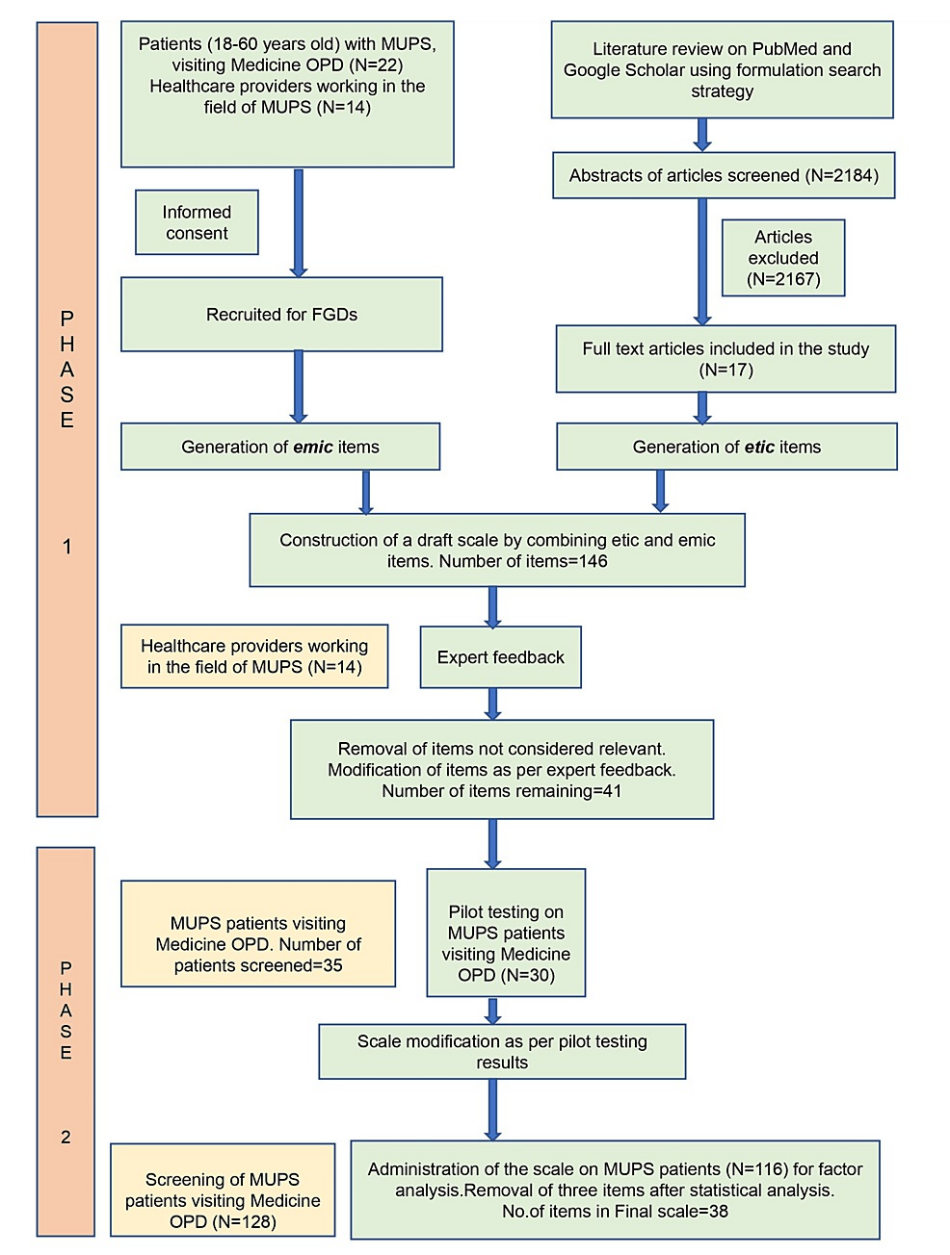
Workflow of the study MUPS, medically unexplained physical symptoms; OPD, out-patient department; FGDs, focus group discussions

A mixed-method study design was used to develop and validate a symptom scale for patients with MUPS. The study was conducted after prior approval from the institute ethics committee (IEC PG- 293/22.07.2020). The workflow of the study is given in Figure 1.

The process was divided into the following two phases:

Phase 1: development of the construct of scale

Step 1: Literature Search and Collection of a Pool of “Etic” Items

(Etic items refer to universal, culturally impartial components, concepts, or phenomena that apply across different cultures or contexts.)

The literature search was done using PubMed and Google through a combination of search terms such as “somatoform disorder,” “somatic symptom disorder,” “functional somatic symptoms,” “medically unexplained symptoms,” “medically unexplained physical symptoms,” questionnaire, scale, and tool. Relevant articles were screened through their titles and abstracts, and items were generated after carefully reading them.

Step 2: Generation of “Emic” Items or the Local Idioms Through Focus Group Discussions

(Emic items represent culturally specific elements, perceptions, or interpretations unique to a particular culture, context, or group of people.)

Eleven focus group discussions (FGDs) were carried out involving patients with MUPS, their caregivers, and healthcare workers (HCWs). A total of 36 participants, including 12 patients, 10 caregivers, and 14 HCWs, were included. The sessions were recorded and transcribed verbatim, and important points were identified by the experts (PR and AS) from the transcriptions and coded. The codes identified were classified based on the underlying theme and sub-theme, and additional items were added to the item pool. The final list of items was then used to formulate the initial construct of the scale, which was simultaneously translated into Hindi language by the language expert.

Phase 2: validation of the scale

Step 3: Content and Face Validation

After the preparation of a draft of the scale, face and content validity was checked through feedback from 15 experts from premier institutes and hospitals of the country working in the field of MUPS. The content validity of the developed instrument was assessed quantitatively through the calculation of the content validity ratio (CVR) and content validity index (CVI). The critical value of CVR and CVI depends on the total number of participants and is determined using Lawshe’s table. The formula for calculating the CVR is CVR = (Ne − N/2)/(N/2), where Ne is the number of participants who consider an item essential, and N is the total number of participants. Thus, all items with a CVR greater than 0.6 were included in the scale, and the remaining items were reviewed and retained if they were felt to be important [5].

The questionnaire was then pre-tested on 30 participants from the OPD in their preferred language, and they were asked to mark the questions as clear (1) or unclear (0). The required changes were made in the draft of the questionnaire, such as the reduction of technical terms and incorporation of more user-friendly terms to increase the ease of the participants filling out the questionnaire.

Step 4: Construct Validation and Assessment of Reliability

One hundred sixteen (N = 116) participants diagnosed with MUPS were informed about the study, and written informed consent was obtained. Data pertaining to socio-demographics and patient history were also recorded. Psychiatric comorbidities were excluded using MINI. Thereafter, PHQ-15 was administered. Subsequently, the newly developed MUPS symptom scale was given to participants to obtain their responses.

Statistical analysis

The obtained data were analyzed using IBM SPSS Statistics, version 28.0 (IBM Corp., Armonk, NY). Quantitative variables were expressed as mean and standard deviation for normally distributed data and median with interquartile range in case of skewed data. Categorical variables were expressed as frequencies and proportions. Kaiser-Meyer-Olkin test and Bartlett’s test of sphericity were used to ensure that the sample size was adequate for factor analysis. Exploratory factor analysis (EFA) with a varimax rotation and Kaiser normalization was used to identify domains and establish construct validity. For determining reliability, Cronbach’s alpha was used. For calculating the degree of correlation between scores on our scale and PHQ-15 scores, Pearson’s correlation was calculated. 

## Results

Socio-demographic characteristics

The mean age of participants was 38.2 ± 9.74 years, with a predominance of females. The duration of symptoms among patients was highly variable, with a range of 1-240 months, and the mean and median duration of symptoms were 29.2 and 24 months, respectively. The detailed socio-demographic characteristics are given in Table 1.

**Table 1:** Socio-demographic characteristics of the participants

Characteristics	Value
Age years (mean ± SD)	38.2 ± 9.74
Gender, N (%)	
Males	34 (29%)
Females	82 (71%)
Marital status, N (%)	
Married	99 (85%)
Unmarried	14 (12%)
Widowed	2 (2%)
Separated	1 (1%)
Place of residence, N (%)	
Urban	82 (71%)
Rural	34 (29%)
Duration of illness in months (mean ± SD)	29.2 ± 31.2

Disease severity

Patient Health Questionnaire-15 (PHQ-15) [6] was used to assess the severity of somatic symptoms experienced by the patients. Each symptom was rated on a scale of 0-2 based on how much the patient was bothered by that symptom. Based on the total score obtained by adding the score for each symptom, patients were classified as having minimal, low, medium, or high severity of somatic symptoms.

Most of the patients included in this study (nearly 50%) had a high severity of somatic symptoms as per PHQ-15 scores. The mean and median PHQ-15 scores were 15.7 ± 6.0 and 15, respectively.

The construct of the questionnaire

Articles about the development, assessment, or review of somatic symptoms or somatization questionnaires were chosen. A total of 17 articles were identified after a comprehensive literature search. The reviewed scales had a variable number of items ranging from 6 to 53. The domains assessed by the scales ranged from 3 to 9. Twelve of the scales were self-report questionnaires, whereas four were interview-based. Only one scale (Brief Symptom Inventory) had both self-report and interview response formats. The different scales covered different time frames ranging from one week to six months, as presented in Table 2. 

**Table 2:** Salient features of existing scales available for the diagnosis of MUPS/somatic symptom/somatoform disorder MUPS, medically unexplained physical symptoms

S. no.	Name of the tool	Items and language	Response format	Time frame	Psychometric properties	Availability in Asian language
1.	Scale for Assessment of Somatic Symptoms (SASS) [7]	20 items with 4 subscales; English	Interview; each item was rated on a scale of 0-3: 0, absent; 1, mild; 2, moderate; 3, severe	2 weeks	Internal consistency (Cronbach’s alpha) = 0.982 (test) and 0.840 (retest); Kendall’s tau B for test-retest > 0.4 for all items except two items that were not scored.	No
2.	Patient Health Questionnaire-15 (PHQ-15) [6]	15 items; English	Self-report; each item is rated on a 3-point scale: 0, not bothered at all; 1, bothered a little; 2, bothered a lot	4 weeks	Cronbach’s alpha = 0.80; higher scores correlate with a greater number of disability days, symptom-related difficulty, and clinic visits.	No
3.	Somatic Symptom Scale-8 (SSS-8) [8]	8 items; English and German	Self-report; 5-point response format ranging from 0 to 4	1 week	Cronbach’s alpha = 0.81; confirmatory factor analysis: an overarching somatic symptom factor and four clusters of symptoms: gastrointestinal, cardiopulmonary, fatigue, and pain. SSS-8 scores correlate strongly with PHQ-2 depression score and GAD-2 anxiety score.	Yes; Japanese, Chinese, & Korean
4.	Bradford Somatic Inventory [9]	46 items (2 for men only); English & Urdu	Self-report; three-choice response format: absent, present on less than 15 days during the past month, and present on more than 15 days during the past month	1 month	Linguistic equivalence of English and Urdu versions: median percentage agreement among the 46 items was 92.5% between English and Urdu responses. Principal components analysis yielded an 8-factor structure that explained 53% of the total variance.	Yes; Urdu
5.	Screening for Somatoform Symptoms-7 [10]	53 items (5 for women only and 1 for men only); English	Self-report; each item is rated on a 4-point scale; range: 0, not at all; 4, very severe	2 years	Cronbach alpha = 0.92; both indices (number of symptoms and severity index) decreased significantly post-treatment. The test-retest correlation coefficient of 0.76 for symptom count and 0.71 for severity index.	No
6.	Depression and Somatic Symptom Scale (DSSS) [11]	22 (12 in depression subscale (DS) + 10 in somatic subscale (SS) including 5 pain items that constitute pain subscale (PS); English	Self-report; each symptom is rated on a scale from 0 to 3: 0, absent; 1, mild; 2, moderate; 3, severe	1 week	Cronbach alpha: DSSS and SS were >0.8. Test-retest reliability: there is a good correlation between scores at the index visit and after one week (Pearson’s correlation coefficient, 0.92). Treatment sensitivity: scores on DSSS decreased significantly post-treatment.	No
7.	Ghent Multidimensional Questionnaire [12]	18 items; English, Dutch, & Turkish	Self-report; the frequency of each item is rated on an 8-point Likert-type scale: 0, never; 1, extremely rarely; 2, rarely; 3, from time to time; 4, regularly; 5, often; 6, very often; 7, constantly	4 weeks	Confirmatory factor analysis confirmed a model consisting of five factors, divided into five symptom groups: pain symptoms (3 items), cardiorespiratory symptoms (4 items), gastrointestinal symptoms (4 items), thermoregulation (3 items), and fatigue (4 items). Cronbach’s alpha of the total scale was higher than 0.90.	No
8.	Somatoform Disorder Schedule (SDS) [13]	53 items; symptoms are grouped into 3 sections: somatization, hypochondriasis, and neurasthenia; English	Interview	-	Good test-retest reliability. Kappa values for most of the symptoms were in the “fair to good” range. One-third of SDS Questions had high inter-rater reliability (Kappa ≥ 0.60).	Yes; Kannada
9.	Schedule for Evaluating Persistent Symptoms (SEPS) [14]	9 items; English	Self-report	-	Cronbach’s α = 0.64; construct validity: ROC curve analysis gave an AUC of 0.63 (95% CI = 0.55–0.72). A cut-off score ≥ 14 indicated pathological MUPS were present with Sn = 65%, PPV = 20%, and NPV = 90%. Exploratory factor analysis revealed two main factors: focus on symptoms and their attribution.	No
10.	Somatic Symptom Index (SSI) [15]	37 items that form part of DSM III diagnostic criteria for somatoform disorder; English & Spanish	Interview	Lifetime	The presence of ≥4 symptoms in males and ≥6 in females was used as the cut-off for the diagnosis of SSI.	No
11.	Somatic Symptom Disorder–B Criteria Scale (SSD-12) [16]	12 items; 3 subscales (cognitive, affective and behavioural) with 4 items each; English & German	Self-report	6 months	Cronbach alpha 0.95 for 12 item scale; factor analysis: different aspects of the B criterion organized into three sub-criteria. Construct validity: SSD-12 total score had a good correlation with PHQ-15 score and Whitely index-7 (a measure of health anxiety). Sensitivity to change: scores were significantly lower in patients with marked improvement at the time of discharge.	Yes; Chinese
12.	4-Dimensional Symptom Questionnaire (4DSQ)- Somatization subscale [17]	16 items; English & Dutch	Self-report; 3-point response scale: 0, absent; 1, doubtfully present; 2, present at a clinically significant level	7 days	Cronbach’s alpha for somatization subscale = 0.80; confirmatory factor analysis showed that a 4-factor model fit best, representing the 4 dimensions of depression, distress, somatization, and anxiety. Construct validity was assessed using (partial) correlations with job stress, coping, and strain.	No
13.	Symptom Checklist-90 R (SCL-90-R)- Somatization subscale [18]	90 items (12 items in somatization subscale); English, Ukrainian, & Russian	Self-report; how bothered are they as a result of each symptom? 0, not at all; 1, a little bit; 2, moderately; 3, quite a bit; 4, extremely	7 days	Cronbach’s alpha for somatization subscale = 0.90, 0.89, and 0.93 in three separate studies. Significant correlation between different dimensions of the scale. Confirmatory factor analysis supports a 9-factor model.	Yes; Japanese, Chinese, Korean, Vietnamese, Hebrew, & Arabic
14.	Brief Symptom Inventory-53 (BSI-53) Somatization subscale [19]	53 items; 7 items in the somatization subscale; English	Self-report or interviewer delivered; how bothered are they as a result of each symptom? 0, not at all; 1, a little bit; 2, moderately; 3, quite a bit; 4, extremely	7 days	All subscales had good reliability, with psychoticism and depression subscales having the lowest and highest reliability, respectively (0.71 for psychoticism and 0.85 for depression). The test-retest reliability range was greatest for the phobic anxiety subscale (0.91). The scale had good correlations with BSI and SCL-R-90 scales (correlation coefficients, 0.92-0.99, respectively).	No
15.	Brief Symptom Inventory-18 (BSI-18) Somatization subscale [20]	18 items; 6 items in somatization subscale; English & Spanish	Self-report; how bothered are they as a result of each symptom? 0, not at all; 1, a little bit; 2, moderately; 3, quite a bit; 4, extremely	7 days	Cronbach’s alpha for somatization subscale = 0.817 in a population of Central American Immigrants. Principal components analysis showed a unidimensional structure for the entire scale, corresponding to psychological distress.	No
16.	Symptom Questionnaire 48 (SQ-48) [21]	48 items; 7 items in somatization subscale; English & Dutch	Self-report; each item is rated on a 5-point Likert scale: 0, never; 1, rarely; 2, sometimes; 3, often; 4, very often	7 days	Cronbach’s alpha: 0.97, complete scale; 0.89, somatization subscale; Somatization subscale showed strong correlation with anxiety (rho = 0.59) and depression subscales (rho = 0.52). The cut-off score for the somatization subscale as per ROC analysis was 1.5, with a sensitivity of 72% and specificity of 66%.	No
17.	Swartz Somatization Index (SSI) [22]	11 items; English	Interview	Lifetime	97.6% of the patients with somatization disorder were correctly identified when at least 5 out of 11 symptoms were present, while the presence of a minimum of 5 symptoms wrongly identified 150 patients (correct negative correspondence, 99%; total error rate, 1.0%).	No

Some scales included symptoms occurring over the patient’s lifetime. A total of 163 symptoms were identified, out of which 130 items remained after removing items that were common across different scales. 

Eleven FGDs involving patients, caregivers, and healthcare professionals were conducted through both online and offline platforms. The discussions were guided by using semi-structured questions. Thematic analysis was conducted using QSR Nvivo software, resulting in the identification of eight themes, as detailed in Table 3. This led to the addition of 16 new items, which were integrated into the existing list of 130 items, bringing the total number of items to 146.

**Table 3:** Summarizing the results of FGD with patients, their caregivers, and healthcare providers FGDs, focus group discussions; MUPS, medically unexplained physical symptoms

S. no.	Theme/subthemes identified	Number of codes generated
1.	Symptoms	
	Pain related symptoms	14
	Non-specific somatic symptoms	10
	Gastrointestinal symptoms	7
	Somatic sensory symptoms	7
	Psychological symptoms	7
	Respiratory symptoms	6
	Cardiovascular symptoms	3
	Biological function-related symptoms	3
2.	Duration of symptoms	9
3.	Impact on life	
	Psychological impact	18
	Impact on functioning	17
	Impact on domestic life	6
	Socio-economic impact	2
	Miscellaneous	1
4.	The course of illness and response to treatment	18
5.	Attribution of symptoms	15
6.	Dealing/coping with symptoms	12
7.	Prevalence of MUPS	5
8.	Scale characteristics	
	Severity rating	5
	Symptom duration to be included	5
	Response format	3
	Language	1
	Miscellaneous	1

Psychometric evaluation of the scale

Content Validity

Table 4 displays the CVR and CVI values calculated for the 146 items derived from etic and emic items. The English and Hindi versions of this table can be viewed here: (http://dx.doi.org/10.13140/RG.2.2.12909.81123). A critical threshold value of CVR was found to be 0.64. All items surpassing a CVR of 0.6 (35 in number) were included in the scale, and the remaining items were reviewed and retained as they were considered necessary by the experts (six in number). Hence, 41 items were considered for review.

**Table 4:** Calculated CVR and CVI for each item as per expert feedback CVR, content validity ratio; CVI, content validity index

S. no.	Item	N_e_	CVI of item	CVR of item
1	Heaviness of head	15	100	1
2	Whole body ache	15	100	1
3	Excessive gas formation	15	100	1
4	Bloating	15	100	1
5	Feeling of tingling or pins and needles sensation in the entire body or a part of it	14	93.3	0.86
6	Pressure/tightness in chest or heart	14	93.3	0.86
7	Feeling of pressure inside head	14	93.3	0.86
8	Burning sensation in chest or stomach	14	93.3	0.86
9	Lack of energy/weakness	14	93.3	0.86
10	Tiredness/lethargy	14	93.3	0.86
11	Stomach/abdominal pain	14	93.3	0.86
12	Headache	14	93.3	0.86
13	Pain in neck and shoulders	14	93.3	0.86
14	Indigestion	14	93.3	0.86
15	Abdominal discomfort	14	93.3	0.86
16	Burning sensation in the entire body or a part of it	13	86.7	0.73
17	Excessive tiredness upon mild exertion	13	86.7	0.73
18	Fever or feeling feverish	13	86.7	0.73
19	Back pain	13	86.6	0.73
20	Chest pain	13	86.7	0.73
21	Gas going from abdomen to other parts of the body	13	86.7	0.73
22	Burning sensation in stomach	13	86.7	0.73
23	Excessive belching or burping	13	86.7	0.73
24	Stomach discomfort or churning feeling in stomach	13	86.7	0.73
25	Numbness in the entire body or a part of it	12	80	0.6
26	Tension/tightness in head like it was tightly held by someone or something	12	80	0.6
27	Light headedness	12	80	0.6
28	Feeling weak or sinking	12	80	0.6
29	Pain in arms, legs, or joints	12	80	0.6
30	Pain starts in one part of the body and spreads to other parts	12	80	0.6
31	Discomfort in and around precordium	12	80	0.6
32	Chest tightness/heaviness	12	80	0.6
33	Abdominal distension	12	80	0.6
34	Poor quality of sleep	12	80	0.6
35	Nervousness	12	80	0.6
36	Heaviness of whole body	11	73.3	0.46
37	Giddiness/dizziness/fainting	11	73.3	0.46
38	Head spinning	11	73.3	0.46
39	Pain in nerves	11	73.3	0.46
40	Heart beating too hard or too fast	11	73.3	0.46
41	Always worried	11	73.3	0.46
42	Stressed out	11	73.3	0.46
43	Always angry/irritated	11	73.3	0.46
44	Restlessness	11	73.3	0.46
45	Constriction of head as if it was gripped tightly from outside	10	66.7	0.33
46	Loss of feeling in arms or legs	10	66.7	0.33
47	Heartburn	10	66.7	0.33
48	Difficulty recovering from exhaustion	10	66.7	0.33
49	Muscle pain	10	66.7	0.33
50	Shortness of breath	10	66.7	0.33
51	Palpitations	10	66.7	0.33
52	Lack of sleep	10	66.7	0.33
53	Lack of appetite	10	66.7	0.33
54	Sleep disturbance (trouble falling asleep/staying asleep, waking up early)	10	66.7	0.33
55	Does not feel like working	10	66.7	0.33
56	Feeling anxious/unable to relax	10	66.7	0.33
57	Difficulty in breathing even when resting	9	60	0.2
58	Shortness of breath with minimal excursion	9	60	0.2
59	Weakness or faintness in the heart	9	60	0.2
60	Constipation	9	60	0.2
61	Fluttering or a feeling of something moving in your stomach	9	60	0.2
62	Passing semen in urine	9	60	0.2
63	Lack of libido	9	60	0.2
64	Wants to lie down all the time	9	60	0.2
65	Heavy feeling in arms/ legs	8	53.3	0.06
66	Dry mouth/ throat	8	53.3	0.06
67	Choking sensation in throat	8	53.3	0.06
68	Crawling/creeping sensations	8	53.3	0.06
69	Weakness of mind	8	53.4	0.06
70	Loss of consciousness	8	73.3	0.06
71	Nausea	8	53.3	0.06
72	Regurgitation of food	8	53.3	0.06
73	Food intolerance	8	53.3	0.06
74	Erectile/ejaculatory dysfunction	8	53.4	0.06
75	Unable to do daily activities	8	53.3	0.06
76	Does not want to go anywhere	8	53.3	0.06
77	Sinking feeling	8	53.3	0.06
78	Hot/ cold spells	7	46.6	-0.06
79	Tension in neck and shoulders	7	46.6	-0.06
80	Head felt hot or burning	7	46.7	-0.06
81	Foreign body/ pressure sensation in throat	7	46.6	-0.06
82	Feeling of lightness	7	46.7	-0.06
83	Swelling in the entire body or a part of it	7	46.7	-0.06
84	Body feels stiff	7	46.7	-0.06
85	Pain in eyes	7	46.7	-0.06
86	Painful breathing or hyperventilation	7	46.7	-0.06
87	Difficulty swallowing as if there was a lump in throat	7	46.6	-0.06
88	Upset abdomen	7	46.7	-0.06
89	Muscle tension	6	40	-0.2
90	Burning sensation while passing urine	6	40	-0.2
91	Loss of touch or pain sensations	6	40	-0.2
92	Lump in throat	6	40	-0.2
93	Itching sensation	6	40	-0.2
94	Trembling/tremors/shaking /shivering	6	40	-0.2
95	Pain elsewhere	6	40	-0.2
96	Unable to walk	6	40	-0.2
97	Bitter taste in mouth	5	33.3	-0.33
98	Bad taste in mouth	5	33.3	-0.33
99	Ringing/buzzing noise in ears or head	5	33.3	-0.33
100	Excessive sweating	5	33.3	-0.33
101	Palms sweating a lot	5	33.3	-0.33
102	Passing urine more frequently	5	33.3	-0.33
103	Amnesia (loss of memory)	5	33.3	-0.33
104	Trouble walking	5	33.3	-0.33
105	Pain during urination	5	33.3	-0.33
106	Sore throat/throat pain	5	33.3	-0.33
107	Diarrhoea /loose bowels	5	33.3	-0.33
108	Difficulty getting full erection	5	33.3	-0.33
109	Unusual/copious vaginal discharge	5	33.3	-0.33
110	Unpleasant sensations in and around genitalia	4	26.7	-0.46
111	Hiccoughing	4	26.7	-0.46
112	Flushing/blushing	4	26.7	-0.46
113	Seizures/convulsions	4	26.7	-0.46
114	Hot/cold sweats	4	26.7	-0.46
115	A part of the body stops working sometimes	4	26.7	-0.46
116	Cough	4	26.7	-0.46
117	Excessively coated tongue	4	26.7	-0.46
118	Sexual indifference	4	26.7	-0.46
119	Burning in eyes	3	20	-0.6
120	Blurred vision	3	20	-0.6
121	Impaired co-ordination or balance	3	20	-0.6
122	Paralysis or localized weakness	3	20	-0.6
123	Pain during sexual intercourse	3	20	-0.6
124	Vomiting (excluding pregnancy)	3	33.3	-0.6
125	Problems during sexual intercourse	3	20	-0.6
126	Sex not pleasurable	3	20	-0.6
127	Other sexual difficulties	3	20	-0.6
128	Blindness	2	13.3	-0.73
129	Spots in front of eyes	2	13.3	-0.73
130	Urinary retention	2	13.3	-0.73
131	Inability to urinate	2	13.3	-0.73
132	Blotchiness or dis-colouration of skin	2	13.3	-0.73
133	Aphonia or loss of voice	2	13.3	-0.73
134	Menstrual cramps	2	13.3	-0.73
135	Anal pain	2	13.3	-0.73
136	Butterflies in stomach	2	13.3	-0.73
137	Irregular menstruation	2	13.3	-0.73
138	Excessive menstrual bleeding	2	13.3	-0.73
139	Darkness/mist in front of eyes	1	6.7	-0.86
140	Double vision	1	6.7	-0.86
141	Deafness	1	6.7	-0.86
142	Hallucinations	1	6.7	-0.86
143	Discharge of fluid from anus	1	6.7	-0.86
144	Continuous/frequent vomiting during pregnancy	1	6.7	-0.86
145	Problems with periods	1	6.7	-0.86
146	Gooseflesh	0	0	-1

Face Validity

Face validation was done through expert feedback and pilot testing on a representative sample of the target population of 30 participants. This method of using both expert judges and the target population is considered to be ideal. One item was eliminated during the pilot testing phase due to redundancy with another item in the scale, resulting in 40 items under review.

Construct Validity

The construct validity of the newly developed scale was assessed by comparing its scores with those of the PHQ-15, a well-established measure for gauging somatic symptom severity. The scale exhibited a moderately strong correlation, with a Pearson correlation coefficient of 0.568. Notably, the correlation between our scale and PHQ-15 scores was significantly higher than that of the SSD-12 subscale (a correlation coefficient of 0.47 with PHQ-15) [16].

In our study, EFA with a varimax rotation and Kaiser normalization was utilized to determine the scale’s factor structure, differing from other studies that typically employed confirmatory factor analysis. Our EFA revealed an 11-factor structure explaining 68.5% of the total variance. This finding aligns with previous observations during the development of the Bradford Somatic Inventory (BSI), which identified a 13-factor structure explaining 65.8% of the variance. During this process, one item was excluded due to a Kaiser-Meyer-Olkin (KMO) measure below 0.5, indicating inadequate sampling adequacy, leading to the retention of 39 items. After a varimax rotation, one item failed to load onto any factor and was subsequently removed, resulting in a final scale comprising 38 items.

Reliability

The Cronbach’s alpha for the newly developed scale was calculated to be 0.916, indicating good reliability.

The newly devised scale consists of 38 items in English and Hindi and can be accessed through the following link: (http://dx.doi.org/10.13140/RG.2.2.34720.19208). The English version of the scale is shown in Table 5. 

**Table 5:** Newly developed MUPS scale The response of the patient should be noted for the following: 1. If a symptom is experienced by the patient on most of the days in the last three months: yes/no 2. If yes, the severity of the symptom is on a scale of 1-3, where 1 is mild, 2 is moderate, and 3 is severe.

S. no	Symptom
1.	Headache/ heaviness of head/feeling of pressure inside head
2.	Whole body ache
3.	Pain in neck and shoulders
4.	Back pain
5.	Pain in arms, legs or joints
6.	Pain in nerves
7.	Muscle pain
8.	Stomach/abdominal pain
9.	Excessive gas formation
10.	Gas going from abdomen to other parts of the body
11.	Bloating or abdominal discomfort
12.	Indigestion
13.	Excessive belching or burping
14.	Abdominal distension
15.	Chest pain
16.	Pressure/heaviness/tightness/discomfort in chest or heart
17.	Discomfort in and around precordium
18.	Difficulty breathing
19.	Felt as if the heart was beating too hard or too fast
20.	Dry mouth/throat
21.	Pressure/foreign body sensation in throat
22.	Feeling of tingling or pins and needles sensation in the entire body or a part of it
23.	Burning sensation in chest or stomach
24.	Burning sensation in the entire body or a part of it
25.	Numbness in the entire body or a part of it
26.	Heaviness of whole body
27.	To feel as if there is swelling in the entire body or a part of it
28.	Itching sensation
29.	Feeling a sense of imbalance while walking
30.	Light headedness
31.	Giddiness/dizziness/fainting
32.	Spinning of head
33.	Lack of energy/weakness
34.	Tiredness/lethargy/easy fatigability
35.	Feeling feverish
36.	Poor quality of sleep
37.	Nervousness
38.	Unusual/copious vaginal discharge (only for women)

## Discussion

The initial pool of items in the scale was derived via both the deductive method (review of literature and evaluation of existing scales on MUPS) and the inductive method (item generation from the responses of individuals through focused group discussions and key individual interviews). This was done to minimize the chances of any important item being missed out and is a major strength of our study. This is in contrast to the Depression and Somatic Symptom Scale (DSSS) [11], PHQ-15, and Ghent Multidimensional Symptom Questionnaire (GMSQ) [12], all of which are based only on items derived from existing scales. The BSI [9], on the other hand, is based only on symptoms derived from case notes of patients visiting hospitals. This scale has been developed with an interview-based response format. Most of the existing scales have a self-report response format, which is inappropriate in our setting as most of our patients are uneducated. Only the Schwartz Somatization Index [10], Somatic Symptom Index [15], Somatoform Disorders Schedule (SDS) [13], and Scale for Assessment of Somatic Symptoms (SASS) [7] are available in interview-based response format, whereas the Brief Symptom Inventory is available in both interview and self-report response format [22].

The scale has been developed in both Hindi and English languages. To the best of our knowledge, none of the existing scales for the diagnosis or evaluation of MUPS is available in Hindi, which is one of the most commonly spoken languages in India. The scale takes into account medically unexplained symptoms occurring on most of the days in the last three months. Most of the existing scales we reviewed included symptoms over a variable period, ranging from one week to a lifetime. We chose three months because most of our patients report chronic symptoms, and symptoms of a shorter duration are more likely to be organic. Our scale consists of a total of 38 items, which ensures that the scale is comprehensive but, at the same time, is not too lengthy. In comparison, the scales that we reviewed have several symptoms ranging from 8 for Somatic Symptom Scale-8 (SSS-8) [8] to 53 for SDS and Screening for Somatoform Symptoms 7 (SOMS-7) [23]. Our scale requires that the severity of each symptom be rated on a three-point scale corresponding to mild, moderate, and severe. This is similar to the response format used in DASS and SASS. Most of the other scales have used a Likert-type response format with different numbers of points. In conclusion, this newly developed scale is a reliable and valid tool for the Indian population.

The reader may refer to the MD thesis (click URL: http://dx.doi.org/10.13140/RG.2.2.20957.63202) for a comprehensive overview of the methodology employed and the results obtained in developing the MUPS Scale for assessing patient symptoms.

## Conclusions

The newly developed scale is a user-friendly diagnostic scale for Indian patients with MUPS. The scale, available in Hindi and English, is designed to be easily administered, quick, and suitable for Indian patients. The scale demonstrates good internal consistency and validity. It serves as a valuable tool for clinicians, offering accurate diagnosis and appropriate treatment for Indian patients with MUPS.

However, our study has some important limitations, the most important being a relatively small sample size, and the scale needs to be evaluated further on larger samples of patients before it becomes available for widespread use by clinicians. The newly developed scale aims to evaluate symptoms and severity levels in patients with MUPS but lacks validation as a diagnostic questionnaire. Therefore, it cannot be directly compared to validated diagnostic tools. This limitation restricts the scale’s utility for diagnostic purposes and necessitates caution in interpreting its results. Future research should focus on validating the scale to enhance its reliability and validity in clinical settings.
